# Preventability of maternal near miss and mortality in Rwanda: A case series from the University Teaching Hospital of Kigali (CHUK)

**DOI:** 10.1371/journal.pone.0195711

**Published:** 2018-06-26

**Authors:** Christophe Benimana, Maria Small, Stephen Rulisa

**Affiliations:** 1 Department of Obstetrics and Gynecology, School of Medicine and Pharmacy, University of Rwanda, Kigali, Rwanda; 2 Obstetrics and Gynecology Department, Division of Maternal Fetal Medicine, Duke University Medical Center, Durham, North Carolina, United States of America; University of Washington, UNITED STATES

## Abstract

**Objective:**

Assess the primary causes and preventability of maternal near misses (MNM) and mortalities (MM) at the largest tertiary referral hospital in Rwanda, Kigali University Teaching Hospital (CHUK).

**Methods:**

We reviewed records for all women admitted to CHUK with pregnancy-related complications between January 1st, 2015 and December 31st, 2015. All maternal deaths and near misses, based on WHO near miss criteria were reviewed (Appendix A). A committee of physicians actively involved in the care of pregnant women in the obstetric-gynecology department reviewed all maternal near misses/ pregnancy-related deaths to determine the preventability of these outcomes. Preventability was assessed using the Three Delays Model.[1] Descriptive statistics were used to show qualitative and quantitative outcomes of the maternal near miss and mortality.

**Results:**

We identified 121 maternal near miss (MNM) and maternal deaths. The most common causes of maternal near miss and maternal death were sepsis/severe systemic infection (33.9%), postpartum hemorrhage (28.1%), and complications from eclampsia (18.2%)/severe preeclampsia (5.8%)/. In our obstetric population, MNM and deaths occurred in 87.6% and 12.4% respectively. Facility level delays (diagnostic and therapeutic) through human error or mismanagement (provider issues) were the most common preventable factors accounting for 65.3% of preventable maternal near miss and 10.7% maternal deaths, respectively. Lack of supplies, blood, medicines, ICU space, and equipment (system issues) were responsible for 5.8% of preventable maternal near misses and 2.5% of preventable maternal deaths. Delays in seeking care contributed to 22.3% of cases and delays in arrival from home to care facilities resulted in 9.1% of near misses and mortalities. Cesarean delivery was the most common procedure associated with sepsis/death in our population. Previous cesarean delivery (24%) and obstructed/prolonged labor (13.2%) contributed to maternal near miss and mortalities.

**Conclusion:**

The most common preventable causes of MNM and deaths were medical errors, shortage of medical supplies, and lack of patient education/understanding of obstetric emergencies. Reduction in medical errors, improved supply/equipment availability and patient education in early recognition of pregnancy-related danger signs will reduce the majority of delays associated with MNM and mortality in our population.

## Introduction

Maternal mortality remains a public health problem throughout the world, and largely affects low- income countries.

In 2010, the WHO (World Health Organization), UNICEF (United Nations Children’s Fund), UNFPA (United Nations Fund for Population Activities) and the World Bank estimated that worldwide, about 260 women die per 100 000 live births. Most of these deaths occur in Sub-Saharan Africa.[2] The Maternal Mortality Ratio (MMR) of 620 per 100 000 live births is markedly elevated in comparison to Europe where the MMR is 21 maternal deaths per 100 000 live births. [2, 3]

In order to reduce the global maternal mortality, the Millennium Development Goals 5 sought to improve maternal health by reducing MMR by three quarters, between 1990 and 2015.[4]

Although the MMR in Rwanda, remains high substantial achievements have been made. Maternal mortality has steadily decreased from 1,071 deaths per 100,000 live births in 2000, to 210 maternal deaths per 100,000 live births in 2015.[5]

A maternal death is defined as “the death of a woman while pregnant or within 42 days of termination of pregnancy, irrespective of the duration and site of the pregnancy, from any cause related to or aggravated by the pregnancy or its management but not form accidental or incidental causes. A maternal near-miss (MNM) is defined as “a woman who nearly died but survived a complication that occurred during pregnancy, childbirth or within 42 days of termination of pregnancy”. [6] In practice, maternal near misses denote women who survive life-threatening events (i.e. organ dysfunction).[7]

Maternal deaths are considered “the tip of the iceberg” of maternal severe morbidity (MNM).[8] For every woman who dies many more will survive serious complications during pregnancy, delivery, and the puerperium.[9]

Examination of MNM evaluates the quality of obstetric care and may contribute to maternal mortality reduction.[10, 11] Because maternal morbidity may precede maternal deaths, the systematic identification and the study of near miss cases provide further understanding of the determinants of maternal mortality.[10]

In our population, surveillance of MNM and mortality is ongoing.[12, 13] This work examines the preventability of these outcomes in our tertiary care population.

## Methods

We performed a retrospective, observational study from January 1st, 2015 to December 31st, 2015 at University Teaching Hospital of Kigali (CHUK) to assesse the preventability of maternal near misses and mortalities.

All women with pregnancy-related complications admitted to CHUK were eligible for study inclusion.

Patients were identified by healthcare providers and from the obstetrics and gynecology admissions registry to identify diagnoses associated with severe maternal morbidity and mortality.

Patients were included in the study if they met the WHO criteria for ‘near misses’ (Appendix A) based on organ system based dysfunction.[6] The WHO Working Group suggests that the organ dysfunction-based approach is ‘the most promising frame for establishing a standard set of criteria’.[14] Women who were pregnant, in labor, delivered or aborted within 42 days were included in the study. All patients who experienced near misses or mortalities at the time of admission or during their hospitalization in the obstetrics and gynecology department were included. We considered transfusion of two or more units of packets red blood cells instead of five because the majority of patients requiring massive transfusion receive less than 5 units in our setting. In addition to considering uterine infections leading to hysterectomy in the near miss category, we also included uterine debridement and repair to describe a partially necrotic, but viable uterus.

Women who developed conditions unrelated to pregnancy (not during pregnancy or 42 days after termination of pregnancy) were excluded from the study.

Data were extracted from the patient’s medical record by investigators. A data collection form was used to collect the necessary information. Demographic information such as age, gravidity, parity, religion, marital status, occupation, and place of delivery were recorded. Details related to the near miss using the organ system dysfunction-based approach were included. Data were retrieved from patients’ files and collected using Epidata version 3.1 and exported to SPSS version 16.0 for analysis. Relevant descriptive statistics like frequencies and percentages were used demonstrate outcomes for near-miss cases at CHUK. Microsoft Excel was used to generate tables and graphics.

We established a voluntary review committee to determine the preventability of maternal near misses and mortalities. The committee reviewed the summarized, abstracted data from the medical record in order to assign medical diagnoses for maternal near misses and mortalities.

The committee consisted of physicians actively involved in the care of pregnant women, e.g. the obstetric-gynecology department staff (6 attendings/ consultants and residents in obstetrics and gynecology). The committee was responsible for reviewing all maternal near misses and deaths to determine preventability. Preventability was assessed using the Three-delay model for a conceptual framework (Appendix B). If a patient’s near miss or death was attributable to one of these delays, the death was deemed preventable. The committee summarized the events leading to the adverse outcome to determine all factors contributing to the catastrophic event(Appendix B).[1] Decisions were made by consensus. The committee agreed all maternal near misses and deaths due to severe sepsis or systemic infections were considered system based problems and deemed preventable. These system based issues included surgical techniques, management errors, appropriate antibiotic usage/availability, and timely referrals. The investigation of contributors to infection related maternal death and near misses is ongoing in our institution.[12]

Privacy and confidentiality were ensured throughout the study. The protocol was approved by CHUK research & ethics committees and to the Institutional Research Board (IRB) of the College of Medicine and Health Sciences/University of Rwanda (CMHS/UR).

## Results

A total of 121 cases were identified and reviewed. The majority of mothers who either died or experienced near misses were in the 25–34 age group. (52.1%) (Table 1). More than 78% were covered by the community health insurance (Mituelle de santé).[15] Most were referred form hospitals in the eastern province (39.7%) and the City of Kigali (30.6%). Half (53.7%) were Para 1 or 2, and most of them self-described their occupation as either farmers or house-wives (76%).

**Table 1:** Characteristics of mothers experiencing near-misses or mortalities.

		N	(%)
Age category	16–24	35	28.9
	25–34	63	52.1
	≥35	23	19
marital status	Single	24	19.8
	Married	90	74.4
	Divorced	1	0.82
	Unknown	6	4.95
Religion	Catholic	29	24
	Protestant	72	59.5
	Jehovah Witness	4	3.3
	Muslim	7	5.8
	Unknown	9	7.4
Occupation	Farmer/housewife	92	76
	Government officer/private	19	15.7
	Unknown	10	8.3
Parity	Para 0	21	17.4
	Para 1 or 2	65	53.7
	Para ≥ 3	35	28.9
Referred from	East	48	39.7
	West	8	6.6
	North	10	8.3
	South	9	7.4
	From home/private clinic	9	7.4
	City of Kigali	37	30.6
Insurance	Community Health	95	78.5
	Government Employee	10	8.3
	No insurance/Private/other types	16	13.2

The major categories life-threatening conditions are described in Table 2. Of the 121 cases, sepsis was associated with 33.90% of all maternal near misses and deaths. The second major category was post-partum hemorrhage (28.1%). Eclampsia/severe preeclampsia comprised 18.2% and 5.8% of maternal near misses and deaths, respectively. Five women experienced other obstetrical disease/complications (4.1%).

**Table 2:** Principle diagnoses associated with maternal near misses and deaths (number, %).

	PPH	Severe preeclampsia	Eclampsia	Severe systemic infections	Ruptured Uterus
Maternal near miss	28 (23.1%)	7 (5.8%)	19 (15.7%)	33 (27.3%)	11 (9.1%)
Maternal death	6 (5.0%)	0 (0.0%)	3 (2.5%)	8 (6.6%)	1 (0.8%)

Many women experienced multi-organ system dysfunction (Table 3). Table 3 highlights the single or combinations of organ system failures mothers experienced. Of the 121 maternal near misses and deaths, 71.1% had coagulation/hematologic dysfunction. Uterine dysfunction/hysterectomy was associated with 40.5% of maternal near misses and deaths.

**Table 3:** Organ system dysfunction (number, %).

	Maternal Near Miss	Maternal Death	Total
Hematologic Dysfunction/coagulation	73 (60.3%)	13 (10.7%)	86 (71.1%)
Uterine Dysfunction/hysterectomy	40 (33.1%)	9 (7.4%)	49 (40.5%)
Neurologic	26 (21.5%)	6(5.0%)	32 (26.4%)
Respiratory	16 (13.2%)	13 (10.7%)	29 (24.0%)
Renal	2 (1.7%)	4 (3.33%)	6 (5%)

Table 4 gives on overview of conditions associated with maternal near misses and deaths. Prior history of cesarean section was associated with 24% of maternal morbidities and mortalities. Prior cesarean section, in either the current or a previous pregnancy, contributed 20.7% and 3.3% of maternal near misses and maternal deaths, respectively. Sepsis associated with post cesarean section peritonitis was associated with the majority of these cases. Obstructed/prolonged labor (13.2%) was another important condition associated with maternal morbidity and mortality. Approximately 10% of maternal near misses and 3.3% of maternal deaths were associated with obstructed/prolonged labor.

**Table 4:** Comorbidities associated with maternal near miss and death (number, %).

	Maternal Near Miss	Maternal Death	Total
Prior Cesarean Delivery	25 (20.7%)	4 (3.3%)	29 (24.0%)
Obstructed/Prolonged Labor	12 (9.9%)	4 (3.3%)	16 (13.2%)
Malaria	5 (4.1%)	1 (0.8%)	6 (5%)
Placenta abnormality (previa/invasive placenta)	4 (3.3%)	0 (0.0%)	4 (3.3%)
HIV	3 (2.5%)	0 (0.0%)	3 (2.5%)

Overall, 65% of all maternal near misses and 10% of maternal deaths were considered preventable (Table 5). Among the most common causes of maternal near miss and mortality, hemorrhage-related conditions were considered preventable in 47% of cases, infectious causes were considered preventable in 25% of cases and 11% of hypertensive disorders were deemed preventable.

**Table 5:** Overview of preventability of maternal near misses and mortality (N = 121)).

	Not Preventable	Preventable	Total
Pregnancy-related Hemorrhage	10 (8.3%)	37 (47%)	47 (38.8%)
Pregnancy-related Infections	0 (0.0%)	31 (25.6%)	31 (25.6%)
Hypertensive Disorders	15 (12.4%)	14 (11.6%)	29 (24%)
Abortive outcomes	0 (0.0%)	23 (19.0%)	23 (19%)
Other medical/surgical complication	6 (5%)	10 (8.3%)	16 (13.2%)
Maternal Near Miss	27 (23.3%)	79 (65.3%)	106(87.6%)
Maternal Death	2 (1.7%)	13 (10.7%)	15 (12.4%)

Tables 5–7 provide additional information related to preventable factors. Delays within the facility (diagnostic and therapeutic) and human errors or mismanagement, were responsible for 41.30% and 5.80% of preventable near misses and mortalities, respectively. Delayed or lacking supplies (blood, medications) and equipment (e.g. ICU bed) were responsible for 5.8% and 2.5% of preventable maternal near miss and deaths, respectively.

**Table 6:** Preventable factors: The first delay/delay in seeking care (N = 121).

	Failed Recognition of Problem	Delayed arrival from home to first health facility	Delay in decision to seek care	Received care from Traditional Healer
Pregnancy-related Hemorrhage	9 (7.4%)	5 (4.1%)	1 (0.8%)	0 (0.0%)
Pregnancy-related Infections	3 (2.5%)	3 (2.5%)	2 (1.7%)	4 (3.3%)
Hypertensive Disorders	11 (9.1%)	1 (0.8%)	4 (3.3%)	0 (0.0%)
Abortive outcomes	10 (8.3%)	7 (5.8%)	6 (5.0%)	5 (4.1%)
Other medical/surgical complication	2 (1.7%)	2 (1.7%)	1 (0.8%)	1 (0.8%)
Maternal Near Miss (n = 50)	27 (22.3%)	11 (9.1%)	8 (6.6%)	4 (3.3%)
Maternal Death (n = 6)	1 (0.8%)	1 (0.8%)	2 (1.7%)	2 (1.7%)

**Table 7:** Preventable factors/ The ‘third delay’: Diagnostic and therapeutic delays within the facility (N = 121).

	Human Error	Supplies/Equipment Lacking	Total
Pregnancy-related Hemorrhage	30 (24.8%)	2 (1.7%)	47 (38.8%)
Pregnancy-related Infections	24 (19.8%)	3 (2.5%)	31 (25.6%)
Hypertensive Disorders	1 (0.8%)	2 (1.7%)	29 (24%)
Abortive outcomes	10 (8.3%)	2 (1.7%)	23 (19%)
Other medical/surgical complication	6 (5.0%)	1 (0.8%)	16 (13.2%)
Maternal Near Miss	50 (41.3%)	7 (5.8%)	106(87.6%)
Maternal Death	7 (5.8%)	3 (2.5%)	15 (12.4%)

First delay: Delay in seeking care: The majority of first delays resulted from mothers failing to recognize the problem (22.3%). Women with hypertensive disorders represented 9.1% who failed to recognized signs of severity, followed by women who experienced abortive outcomes (abortion or ectopic pregnancy). Another important preventable factor was the delay in seeking care from home (9.1%). Women with abortive outcome (5.8%) delayed to consult from their home. Of these outcomes, 6.6% of maternal near miss and 1.7% of maternal deaths could have been prevented if they made the decision earlier to seek care in the health facility. In our population, 3.3% of women sought care with a traditional healer before seeking care in a health facility.

The second delay (Delay in reaching the correct/tertiary care facility) was not a major obstacle in our population. Lack of transportation and the quality of roads were not identified as barriers to care in this study. Only 0.8% of maternal near misses and maternal deaths were considered preventable due to difficulty reaching the tertiary care facility.

In our study, the third delay (diagnostic and therapeutic delays within the healthcare facility) accounted for 87% of preventable maternal near misses and 12% of preventable maternal deaths.

## Discussion

Severe systemic infection, postpartum hemorrhage, and hypertensive disorders (severe preeclampsia/eclampsia) were the leading conditions associated with MNM and mortality. These findings are consistent with data from similar low—income countries.[16–19]

Prior cesarean delivery and obstructed/prolonged labor were associated with 24% and 13.2% of all MNM and maternal mortality, respectively. These findings are similar to those reported by a study done in Sao Francisco Valley in Brazil. [20]

This study on preventability of MNM and maternal mortality identified important delays in our population. We deemed the majority of these adverse outcomes preventable. The overall preventability of MNM and mortality were 65% and 10%, respectively. The majority (52.1%) of maternal near misses and deaths occurred in the 25-35-year age group. A study from Myanmar, also identified the majority of women with near misses/mortalities in the range of 30–34 years and 35–39 years, however, work from Brazil reported slightly younger women affected by these outcomes. [21, 22] The third delay (diagnostic and therapeutic delays within the healthcare facility) were associated with the majority of preventable near misses (87%). In contrast, only 12% of maternal mortalities were considered preventable through improved management in the tertiary care facility. This is similar to studies from Tanzania and Jordan where poor management and/or inaccurate diagnoses contributed to the majority maternal mortalities (12, 13).[23, 24]

Failure of recognition of the acute problem is one factor that may contribute to facility level delays. In our study, 22.3% of maternal near misses and approximately 1% (0.8%) of maternal deaths were due to failure of recognition of the problem. This contribution to preventability is lower than described by Mohammed et al. in a study from Eastern Sudan (75%).[25] Delay in patient presentation from home to the care facility was culpable in 9.1% and less than 1%(0.8%) of maternal near misses and deaths, respectively. Mohammed *et al*. and Yunus *et al* describe a higher proportion of patients unable to judge the graveness of the disease (45%) in a Pakistani population.[26]

Lack of supplies and equipment (blood products, ICU bed space) were attributed to 5.8% of preventable factors in maternal near misses and 2.5% of preventable maternal mortalities. This finding is similar to reports from Egypt, Malawi, and Bangladesh.[17, 27, 28] Our findings, however, demonstrate a lower impact from the ‘third delay’ than in other settings.[29]

The ‘second delay’ was not a significant contributor to MNM and death in our population. Less than 1% of maternal deaths and near misses were attributed to delayed referrals to the tertiary health facility. Roads and transportation were not considered major determinants of preventable MNM and death in this study. These findings demonstrate a lower contribution to adverse outcomes from the “second delay” than in other studies.[24] Patients in Rwanda requiring emergency tertiary care services are typically transported in ambulances covered by community insurance. This structure may reduce the impact of roads and transportation on maternal near misses and mortalities.

Insurance status/religion are socio-demographic factors of possible importance. In addition to occupation, these demographic factors reflect population characteristics. The study population demonstrated socio-demographic characteristics similar to the majority of patients in our population. Similar to the general obgyn population, the majority of women had access to health insurance.

The study is limited by the retrospective design and dependence on information recorded in the patient record. A prospective component would help address incomplete information. A qualitative component aimed at patients or their surviving family members would be useful to determine whether patients experienced multiple, unmeasured contributors to their adverse outcomes. This type of analysis could potentially enable patients and family members express their perception of solutions to ameliorate delays.

## Conclusion

This work highlights the importance of strengthening health systems; providers and patient education to further reduce maternal near misses and deaths. Unlike other work, the Delay in reaching the tertiary care facility (second delay) was not a major factor in this study. Increased effort should be made to educate pregnant women, and to reduce deficiencies in key emergency medical supplies. Doctors and health providers in district hospitals should continue to receive continuing education supervision, and feedback from referral hospitals. The high numbers of sepsis cases reflect multiple systems based-problems (e.g. sterilization, surgical technique/inexperience, overuse of antibiotics/multidrug resistance, postoperative management) The specific causes and preventability of post-cesarean sepsis are still not clear in our population. Further research and surveillance in this area is ongoing.

## Appendix A: Near-miss criteria [6]

**Cardiovascular**
  - Shock, cardiac arrest (absence of pulse/heart beat and loss of consciousness), severe hypoperfusion (lactate >5 mmol/l or >45 mg/dl), severe acidosis (PH< 7.1), use of continuous vasoactive drugs, cardiopulmonary resuscitation.
**Respiratory**
  - Acute cyanosis, gasping, severe tachypnea (respiratory rate >40 breaths per minute), severe bradypnea (respiratory rate <6 breaths per minute), severe hypoxemia (O2 saturation <90% for ≥60 minutes or PAO2/FiO2 <200), intubation and ventilation not related to anesthesia,
**Renal**
  - Oliguria non-responsive to fluids or diuretics, severe acute azotemia (creatinine ≥300 μmol/ml or ≥3.5 mg/dl, dialysis for acute renal failure/referred for dialysis
**Hematologic/coagulation**
  - Failure to form clots, severe acute thrombocytopenia (<50 000 platelets/ml), massive transfusion of blood or red cells (≥2 units)
**Hepatic**
  - Jaundice in the presence of pre-eclampsia, severe acute hyperbilirubinemia (bilirubin >100 μmol/l or >6.0 mg/dl)
**Neurologic**
  - Prolonged unconsciousness (lasting ≥12 hours)/coma (including metabolic coma), stroke, uncontrollable fits/status epilepticus, total paralysis.
**Alternative severe proxy**
  - Hemorrhage or infection leading to hysterectomy/repair, uterine infection leading to uterine debridement and repair.

## Appendix B: The Three delays model [1]

1. **Delay in seeking care**
  1a) Harmful traditional practice
  1b) Family poverty
  1c) Failure of recognition of the problem
  1d) Lack of the decision to go to health facility
  1e) Delayed referral from home
2. **Delay in reaching at right facility**
  2a) Delayed arrival to referral facility
  2b) Lack of roads
  2c) Lack of transportation
  2d) No facility within reasonable distance
3. **Delay within the facility (diagnostic and therapeutic)**
  3a) Delayed to arrival to next facility from referral from another facility
  3b) Delayed manager after admission
  3c) Delayed or lacking of supplies and equipment
  3d) Human error or mismanagement

## Supporting information

S1 Code Coding for data maternal near miss in Rwanda. (DOCX)

S1 Dataset Data maternal near miss in Rwanda. (XLSX)
