# Supplementary material for: Preventability of maternal near miss and mortality in Rwanda: A case series from the University Teaching Hospital of Kigali (CHUK)
Source: PLoS One. 2018 Jun 26;13(6):e0195711. doi: 10.1371/journal.pone.0195711 (PMC6019403; doi:10.1371/journal.pone.0195711)
Supplement: S1 Code — (DOCX) [file pone.0195711.s001.docx]

**Annex: table 1: near-miss criteria**

| **Life-threatening conditions (near-miss criteria)** | | | |
| --- | --- | --- | --- |
| **Dysfunctional systems** | **Clinical criteria** | **Laboratory markers** | **Management based proxy** |
| ***Cardiovascular*** | - Shock, - Cardiac arrest (absence of pulse/heart beat and loss of consciousness) | - Severe hypoperfusion (lactate >5 mmol/l or >45 mg/dl), - severe acidosis (PH< 7.1) | - use of continuous vasoactive drugs, - cardiopulmonary resuscitation |
| ***Respiratory*** | - Acute cyanosis, - gasping, - severe tachypnea (respiratory rate >40 breaths per minute), - severe bradypnea (respiratory rate <6 breaths per minute) | - severe hypoxemia (O2 saturation <90% for ≥60 minutes or PAO2/FiO2 <200) | - intubation and ventilation not related to anaesthesia, |
| ***Renal*** | - Oliguria non-responsive to fluids or diuretics, | - Severe acute azotemia (creatinine ≥300 μmol/ml or ≥3.5 mg/dl | - dialysis for acute renal failure/referred for dialysis |
| ***Hematologic/coagulation*** | - Failure to form clots | - severe acute thrombocytopenia (<50 000 platelets/ml) | - massive transfusion of blood or red cells (≥2 units) |
| ***Hepatic*** | - Jaundice in the presence of pre-eclampsia, | - severe acute hyperbilirubinemia (bilirubin >100 μmol/l or >6.0 mg/dl) |  |
| ***Neurologic*** | - Prolonged unconsciousness (lasting ≥12 hours)/coma (including metabolic coma), - stroke, - uncontrollable fits/status epilepticus, - total paralysis |  |  |
| ***Alternative severe proxy*** |  |  | - Uterine hemorrhage or infection leading to hysterectomy |
| ***Maternal vital status*** | Maternal death | | |

**Annex: table 2: data collection form**

| **MATERNAL NEAR MISS TOOL: INDIVIDUAL DATA COLLECTION FORM** | | | |
| --- | --- | --- | --- |
| ID: | | | |
| **SCREENING QUESTIONS** | | | |
| **Age of the patient** | **encircle appropriate response** | | |
| < 20 years old | 1 |  | |
| 20-35 years old 32 yr old | 2 |  | |
| >35 years old | 3 |  | |
| Unknown age | 4 |  | |
| **Marital status** | **encircle appropriate response** | | |
| single | 1 |  | |
| married | 2 |  | |
| divorced | 3 |  | |
| Widowed | 4 |  | |
| Other/unknown | 5 |  | |
| **Religion** | **encircle appropriate response** | | |
| Catholic | 1 |  | |
| Protestant | 2 |  | |
| Jehovah witness | 3 |  | |
| Muslim | 4 |  | |
| Other | 5 |  | |
| Unknown | 6 |  | |
| **Occupation** | **encircle appropriate response** | | |
| Farmer/laborer/housewife | 1 |  | |
| Government officer/private | 2 |  | |
| Unknown | 3 |  | |
| **Referred from** | | | |
| EAST province | 1 |  | |
| WEST province | 2 |  | |
| NORTH province | 3 |  | |
| SOUTH province | 4 |  | |
| From home/private clinic | 5 |  | |
| City of Kigali | 6 |  | |
|  |  |  | |
| **Parity** | **encircle appropriate response** | | |
| 0 | 1 |  | |
| 1-2 | 2 |  | |
| ≥ 3 | 3 |  | |
|  | | | |
| Yes |  |  | |
| No |  |  | |
| **In the questions A to E, please specify:**  **0= The condition is NOT present.**  **1= The condition is present.** | | | |
| **Severe complications / potentially life-threatening conditions** | | | **Response** |
| A. Severe postpartum hemorrhage | | |  |
| B. Severe preeclampsia | | |  |
| C. Eclampsia | | |  |
| D. Sepsis or severe systemic infection | | |  |
| E. Ruptured uterus | | |  |
| **Organ dysfunction / life-threatening conditions** | | | |
| *C0 Cardiovascular dysfunction*:  [shock, use of continuous vasoactive drugs, cardiac arrest,  resuscitation, severe hypoperfusion (lactate >5 mmol/L or >45mg/dL) or severe acidosis (pH<7.1)] | | |  |
| *C1 Respiratory dysfunction:*  [acute cyanosis, gasping, severe tachypnea (respiratory rate>40 bpm), severe bradypnea (respiratory rate<6 bpm), severe hypoxemia (PAO2/FiO2<200 O2 saturation <90% for ­60min) or intubation and ventilation not related to anaesthesia] | | |  |
| *C2 Renal dysfunction:*  [oliguria non responsive to fluids or diuretics, dialysis for acute renal failure or severe acute azotemia (creatinine ­300umol/ml or ­3.5mg/dL)] | | |  |
| *C3 Coagulation/hematologic dysfunction:*  [failure to form clots, massive transfusion of blood (­ 2 units) or severe acute thrombocytopenia (<50,000 platelets/ml)] | | |  |
| *C4 Hepatic dysfunction:*  [jaundice in the presence of pre-eclampsia, severe acute hyperbilirubinemia (bilirubin>100umol/L or >6.0mg/dL)] | | |  |
| *C5 Neurologic dysfunction:*  [prolonged unconsciousness / coma (lasting >12 hours), stroke, status epilepticus / uncontrollable fits or global paralysis] | | |  |
| *C6 Uterine dysfunction / Hysterectomy:*  [haemorrhage or infection leading to hysterectomy, uterine infection leading to uterine debridement and repair] | | |  |
| **Maternal deaths** | | |  |
| Death during pregnancy or within 42 days of termination of pregnancy | | |  |
| Death after 42 days of termination of pregnancy | | |  |
| **MATERNAL INFORMATION** | | | |
|  | | | |
| **Final mode of delivery / end of pregnancy. Please specify:** | | |  |
| 1= Vaginal Delivery | | |  |
| 2= Caesarean section | | |  |
| 3= Abortion | | |  |
| 4= Laparotomy for ectopic pregnancy | | |  |
| 5= Laparotomy for ruptured uterus | | |  |
| 6= Women discharged or died still pregnant | | |  |
| 7= Unknown / other | | |  |
| **UNDERLYING CAUSES OF DEATH / NEAR MISS** | | | |
| **Please specify: (0=No 1=Yes)** | | | |
| Pregnancy with abortive outcome (abortion/ectopic pregnancy) | | |  |
| Obstetric hemorrhage | | |  |
| Hypertensive disorders | | |  |
| Pregnancy-related infection | | |  |
| Other obstetric disease or complication | | |  |
| Medical/surgical/mental disease or complication | | |  |
| Unanticipated complications of management | | |  |
| Coincidental conditions | | |  |
| Unknown | | |  |
| **CONTRIBUTORY / ASSOCIATED CONDITIONS** | | | |
| **Please specify: (0=No 1=Yes)** | | | |
| HIV infection | | |  |
| Previous caesarean section | | |  |
| Prolonged/obstructed labor | | |  |
| Malaria | | |  |
| Unknown | | |  |
| Other condition specified in the local manual of operations | | |  |
| **About the use of interventions, please specify whether the woman received any of the following : (0=No 1=Yes)** | | | |
| **A. Prevention of postpartum haemorrhage** | | | |
| Oxytocin | | |  |
| Other uterotonic | | |  |
| **B. Treatment of postpartum haemorrhage** | | | |
| Ocytocin | | |  |
| Ergometrine | | |  |
| Misoprostol | | |  |
| Other uterotonics | | |  |
| Tranexamic acid | | |  |
| Removal of retained products | | |  |
| Repair of tear/laceration | | |  |
| Vaginal packing | | |  |
| Balloon or condom tamponade | | |  |
| Artery ligation (uterine/hypogastric) | | |  |
| Hysterectomy | | |  |
| Abdominal packing | | |  |
| Uterine repair | | |  |
| **C. Anticonvulsant** | | | |
| Magnesium sulfate | | |  |
| Other anticovulsant | | |  |
| **D. Antibiotics** | | | |
| Prophylactic antibiotic during caesarean section | | |  |
| Parenteral, therapeutic antibiotics | | |  |
| **Could the maternal near miss/death be preventable? specify:(0=No 1=Yes)** | | |  |
| **If “yes”, specify based on the three delay models** | | |  |
| **1. Delay in seeking care** | | |  |
| 1a) harmful traditional practice | | |  |
| 1b) family poverty | | |  |
| 1c) failure of recognition of the problem | | |  |
| 1d) lack of the decision to go to health facility | | |  |
| 1e) delayed referral from home | | |  |
| **2. Delay in reaching at right facility** | | |  |
| 2a) Delayed arrival to referral facility | | |  |
| 2b) Lack of roads | | |  |
| 2c) Lack of transportation | | |  |
| 2d) No facility within reasonable distance | | |  |
| **3. Delay within the facility (diagnostic and therapeutic)** | | |  |
| 3a) Delayed to arrival to next facility from referral from another facility | | |  |
| 3b) Delayed manager after admission | | |  |
| 3c) Delayed or lacking of supplies and equipment | | |  |
| 3d) Human error or mismanagement | | |  |
